# Shorter pruritus period and milder disease stage are associated with response to nalfurafine hydrochloride in patients with chronic liver disease

**DOI:** 10.1038/s41598-022-11431-1

**Published:** 2022-05-04

**Authors:** Tadamichi Kawano, Masanori Atsukawa, Akihito Tsubota, Noritomo Shimada, Hidenori Toyoda, Koichi Takaguchi, Joji Tani, Asahiro Morishita, Atsushi Hiraoka, Shigeru Mikami, Toru Ishikawa, Hironao Okubo, Tsunamasa Watanabe, Tomomi Okubo, Taeang Arai, Korenobu Hayama, Norio Itokawa, Chisa Kondo, Katsuhiko Iwakiri

**Affiliations:** 1 Division of Gastroenterology and Hepatology, Department of Internal Medicine, Nippon Medical School, 1-1-5, Sendagi, Bunkyo-ku, Tokyo, 113-8603 Japan; 2 Core Research Facilities, The Jikei University School of Medicine, Tokyo, Japan; 3 Department of Internal Medicine, Division of Gastroenterology and Hepatology, Otakanomori Hospital, Kashiwa, Japan; 4 Department of Gastroenterology, Ogaki Municipal Hospital, Ogaki, Japan; 5 Department of Hepatology, Kagawa Prefectural Central Hospital, Takamatsu, Japan; 6 Department of Gastroenterology, Kagawa University Graduate School of Medicine, Kagawa, Japan; 7 Gastroenterology Center, Ehime Prefectural Central Hospital, Matsuyama, Japan; 8 Department of Internal Medicine, Division of Gastroenterology, Kikkoman General Hospital, Noda, Japan; 9 Department of Gastroenterology, Saiseikai Niigata Hospital, Niigata, Japan; 10 Department of Gastroenterology, Juntendo University Nerima Hospital, Tokyo, Japan; 11 Division of Gastroenterology and Hepatology, St. Marianna University School of Medicine, Kawasaki, Japan; 12 Division of Gastroenterology, Department of Internal Medicine, Nippon Medical School Chiba Hokusoh Hospital, Inzai, Japan

**Keywords:** Gastroenterology, Skin diseases

## Abstract

Nalfurafine hydrochloride, a selective κ-opioid receptor agonist has been approved for pruritus in patients with chronic liver disease. However, not all patients respond to nalfurafine hydrochloride. The aim of this study was to clarify the efficacy of nalfurafine hydrochloride. The subjects were patients with chronic liver disease complicated by pruritus who were treated with nalfurafine hydrochloride between May, 2015, and May, 2021. The degree of pruritus was evaluated based on the Visual Analog Scale (VAS) score and the Kawashima’s pruritus score. Nalfurafine hydrochloride 2.5 μg was orally administered once a day for 12 weeks. A decrease in the VAS score of ≥ 25 mm or the Kawashima’s pruritus score of ≥ 1 scores was designated as relevant response**.** The former of ≥ 50 mm or the latter of ≥ 2 scores as remarkable response. The 326 patients who were evaluated the efficacy at 12 weeks. The median time suffering from pruritus to administration of nalfurafine hydrochloride was 4 months. The median VAS score improved from 70.0 mm before administration to 40.0 and 30.0 mm at 4 and 12 weeks of treatment, respectively. On multivariate analysis, shorter itching period and lower FIB-4 index value were extracted as the independent factors related to remarkable responder. On multivariate analysis, shorter itching period was extracted as the only independent factor related to relevant responder. In conclusion, this study suggested nalfurafine hydrochloride treatment markedly improves pruritus in patients with chronic liver disease. A short pruritus period and less-advanced fibrosis were associated with response to nalfurafine hydrochloride.

## Introduction

Pruritus (also known as itch) is defined as an uncomfortable sensation of the skin that provokes a desire to scratch. The characteristics and complaints of pruritus differ from person to person. Some people complain that “itch boiling up from inside the body,” while others complain that “sudden attacks of itching.” Such various pruritic conditions occur not only in patients with skin disease (such as atopic dermatitis and urticaria) but also in patients with diabetes, chronic kidney disease, and chronic liver disease^1^. The prevalence of pruritus in patients with chronic liver disease is approximately 40%^2^, while that in the general population is approximately 8%^3^. Pruritus in many patients with chronic liver disease is caused by cholestasis; however, the degree of pruritus is not always correlated with the grade of cholestasis and serum bile acid concentrations, because it is influenced by multiple factors^4^.

The mechanism underlying pruritus (including itch-related nervous systems) is complex, and not fully elucidated. Peripheral pruritus is considered as an inflammatory reaction and is responsive to conventional treatments (such as antihistamine, antiallergic drugs, moisturizer, and topical steroids). When stimulated by an IgE antibody and complement, mast cells in the skin dermis and subcutaneous adipose tissue release substances (such as histamine and protease), which bind to the itching receptors in the dermoepidermal junction. The itch signals are transmitted to the spinal cord through the C fibers and are finally recognized as pruritus in the cerebrum.

Meanwhile, central pruritus is considered imbalances in the expression and/or activation of the μ- and κ-opioid systems^5^ and is less responsive to conventional treatments. Pruritus is triggered by μ-opioid receptor agonists and suppressed by κ-opioid receptor agonists. Since the κ-opioid receptor has an opposite pharmacological action on the μ-opioid receptor, the κ-opioid receptor activation inhibits the μ-opioid receptor-mediated action. Endogenous opioids (such as β-endorphin and dynorphin) are produced by keratinocytes and nerve cells. β-endorphin (ligand of the μ-opioid receptor) induces pruritus, whereas dynorphin (ligand of the κ-opioid receptor) inhibits pruritus. Under the normal conditions (without itch), the μ- and κ-opioid receptor activities maintain equilibrium.

Patients with chronic liver disease frequently have universal pruritus, which is not or less responsive to conventional treatments largely due to central pruritus. Nalfurafine hydrochloride, a selective κ-opioid receptor agonist, has recently been approved as a new anti-central itch drug for patients with chronic liver disease accompanied by pruritus in Japan, based on the treatment outcomes in a randomized, double-blinded trial^6,7^. This randomized trial demonstrated the efficacy of nalfurafine hydrochloride 2.5 or 5 μg daily for 12 weeks using a visual analogue scale (VAS) and the pruritus score. Another clinical study showed that the response rate to nalfurafine hydrochloride was 71% in 24 refractory pruritus patients^8^. However, these studies excluded patients with severe liver cirrhosis (i.e., Child–Pugh class C) from the subjects, who may have more frequent or more severe pruritus. In addition, they did not evaluate which factors were associated with the effect of nalfurafine hydrochloride.

The aim of this multicenter study was to determine factors associated with the response to nalfurafine hydrochloride in a relatively large, real-world cohort, including patients with severe liver cirrhosis.

## Results

### Characteristics of patients

**Figure 1:**
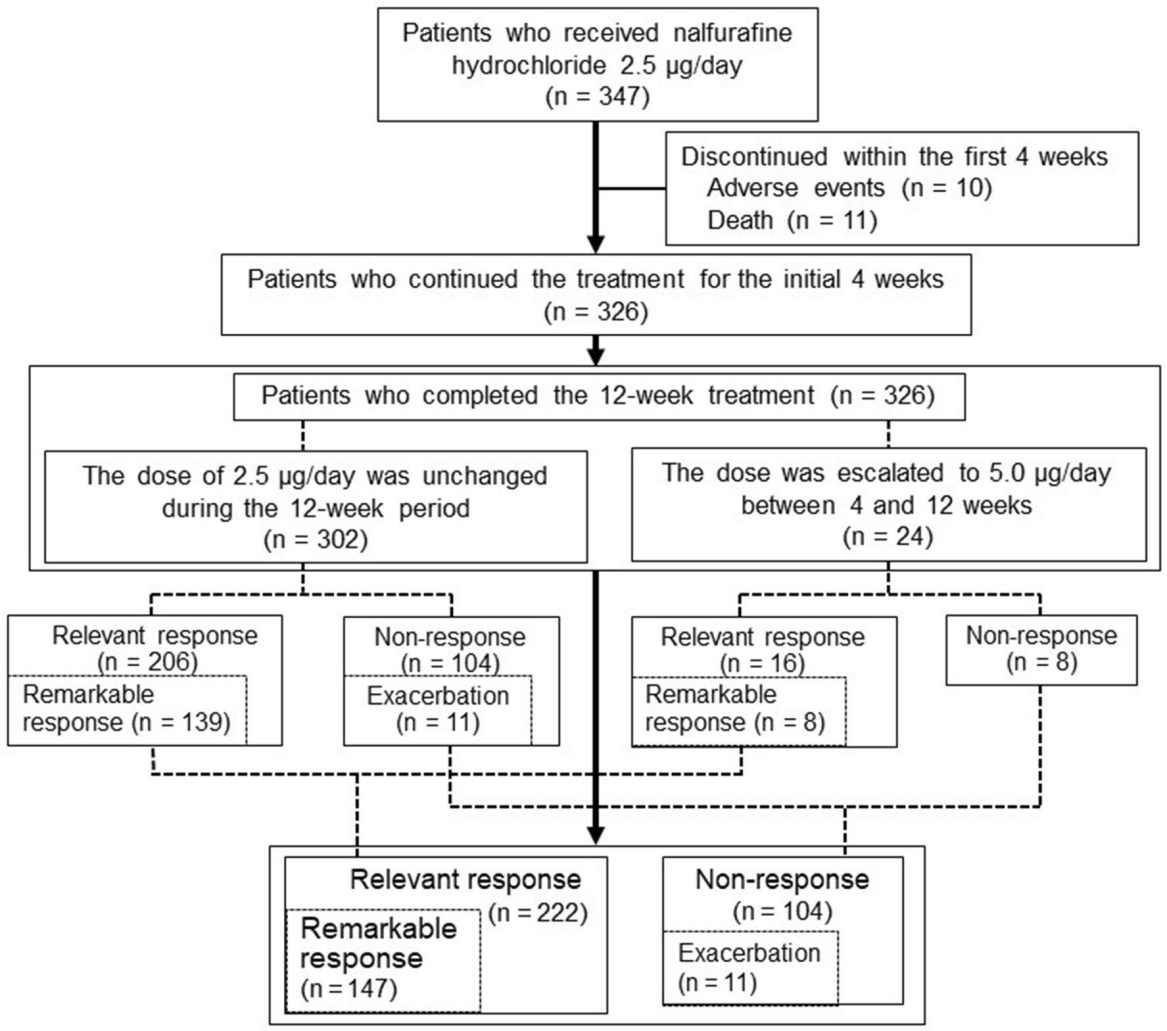
Flow chart of this study.

Of the 347 patients, 326 patients who received nalfurafine hydrochloride as scheduled for 12 weeks without any adverse events (Table 1). The remaining 21 patients discontinued the treatment within the first 4 weeks due to adverse events and were excluded from this analysis (Fig. 1, Supplementary Table 1). The study cohort comprised 164 males and 162 females, with a median age of 71 (range, 18–93) years. The most prevalent etiology of liver disease was chronic hepatitis C (35.0%; 114/326). Of the 326 patients, 192 (58.9%), 84 (25.8%), and 50 (15.3%) had Child–Pugh class A, B, and C, respectively. Sixty (18.4%) patients had a history of hepatocellular carcinoma treatment. The median period from pruritus onset to nalfurafine hydrochloride administration was 4.0 (range, 0.25–120) months.

**Table 1:** Baseline characteristics of the 326 patients.

Factor	
Gender (male/female)	164/162
Age (years)	71 (18–93)
Height (cm)	158 (133–176)
Body weight (kg)	57.2 (29.0–102.5)
Itching period (months)	4.0 (0.25–120)
HCV/PBC/Alc/NASH/HBV/AIH/Overlap/Others	114/62/47/41/21/11/6/24
Child–Pugh classification A/B/C	192/84/50
Hepatocellular carcinoma (presence/absence)	60/266
Ascites (none/mild/moderate)	255/41/30
Hepatic encephalopathy (none/grade I–II/grade III–IV)	284/31/11
Past history of spontaneous bacterial peritonitis (yes/no)	2/324
Past history of variceal bleeding (yes/no)	3/323
White blood cell (/mm^3^)	4800 (1100–21,330)
Hemoglobin (g/dL)	11.7 (6.7–18.7)
Platelet (× 10^3^/mm^3^)	126 (23–549)
PT (%)	80.6 (6.7–173)
Albumin (g/dL)	3.5 (1.5–4.8)
AST (U/L)	37 (3–1177)
ALT (U/L)	25 (2–1509)
Total bilirubin (mg/dL)	1.0 (0.2–29.0)
ALP (U/dL)	391 (75–4600)
γ-GTP (mg/dL)	47 (9–1769)
BUN (mg/dL)	17.0 (4.8–143.2)
Creatinine (mg/dL)	0.84 (0.33–11.7)
eGFR (mL/min/1.73 m^2^)	61.0 (3.9–140.2)
AFP (ng/mL)	4.3 (0.7–180,434)
M2BPGi (C.O.I.)	2.66 (0.42–18.2)
FIB-4 index	3.84 (0.52–31.0)
ALBI score	− 2.17 (− 3.53 to 0.67)
MELD score	11.1 (6–30)

Data are presented as numbers or medians (ranges).

*HCV* hepatitis C virus, *PBC* primary biliary cholangitis, *Alc* alcoholic hepatitis, *NASH* non-alcoholic steatohepatitis, *HBV* hepatitis B virus, *AIH* autoimmune hepatitis, *PT* prothrombin time, *AST* aspartate aminotransferase, *ALT* alanine aminotransferase, *ALP* alkaline phosphatase, *γ-GTP* gamma glutamyl transpeptidase, *BUN* blood urea nitrogen, *eGFR* estimated glomerular filtration rate, *AFP* α-fetoprotein, *M2BPGi* Mac-2 binding protein glycosylation isomer, *FIB-4* fibrosis-4, *ALBI score* albumin–bilirubin score, *MELD score* model for end-stage liver disease score.

### Changes in the degree of pruritus after nalfurafine hydrochloride

The VAS scores and Kawashima’s scores at baseline were moderately correlated (*r* = 0.563, *p* = 3.78 × 10^−8^). The VAS scores of 58.0 ± 14.7 mm, 69.5 ± 13.6 mm, and 81.3 ± 14.3 mm corresponded to the Kawashima’s scores of 2, 3, and 4, respectively (*p* = 6.18 × 10^−14^). The median VAS scores significantly improved from 70.0 mm at baseline to 40.0 and 30.0 mm at 4 and 12 weeks of treatment, respectively (*p* = 1.47 × 10^−21^ and 7.74 × 10^−6^, respectively; Supplementary Fig. 1). No differences in the VAS scores at baseline were noted between different etiologies of chronic liver disease (p = 0.84; Supplementary Fig. 2). The Kawashima’s scores significantly improved at 4 and 12 weeks of treatment in the daytime (*p* = 7.90 × 10^−34^ and 9.05 × 10^−51^, respectively) and nighttime (*p* = 1.83 × 10^−32^ and 2.15 × 10^−46^, respectively; Supplementary Fig. 3).

### Factors associated with remarkable response to nalfurafine hydrochloride

Of the 326 patients who completed the treatment as scheduled, 147 (45.4%) had remarkable response. At baseline, remarkable responders showed a shorter itching period (*p* = 1.29 × 10^−5^), Child–Pugh A or B (*p* = 6.31 × 10^−3^), higher platelet count (*p* = 1.88 × 10^−8^), higher prothrombin time (*p* = 6.68 × 10^−4^), lower aspartate aminotransferase (*p* = 4.02 × 10^−3^), lower total bilirubin (*p* = 2.41 × 10^−2^), lower FIB-4 index (*p* = 4.01 × 10^−8^), and lower ALBI score (*p* = 1.04 × 10^−4^), as compared to non-remarkable responders (Supplementary Table 2). No significant difference was noted in other baseline factors between the two groups.

**Figure 2:**
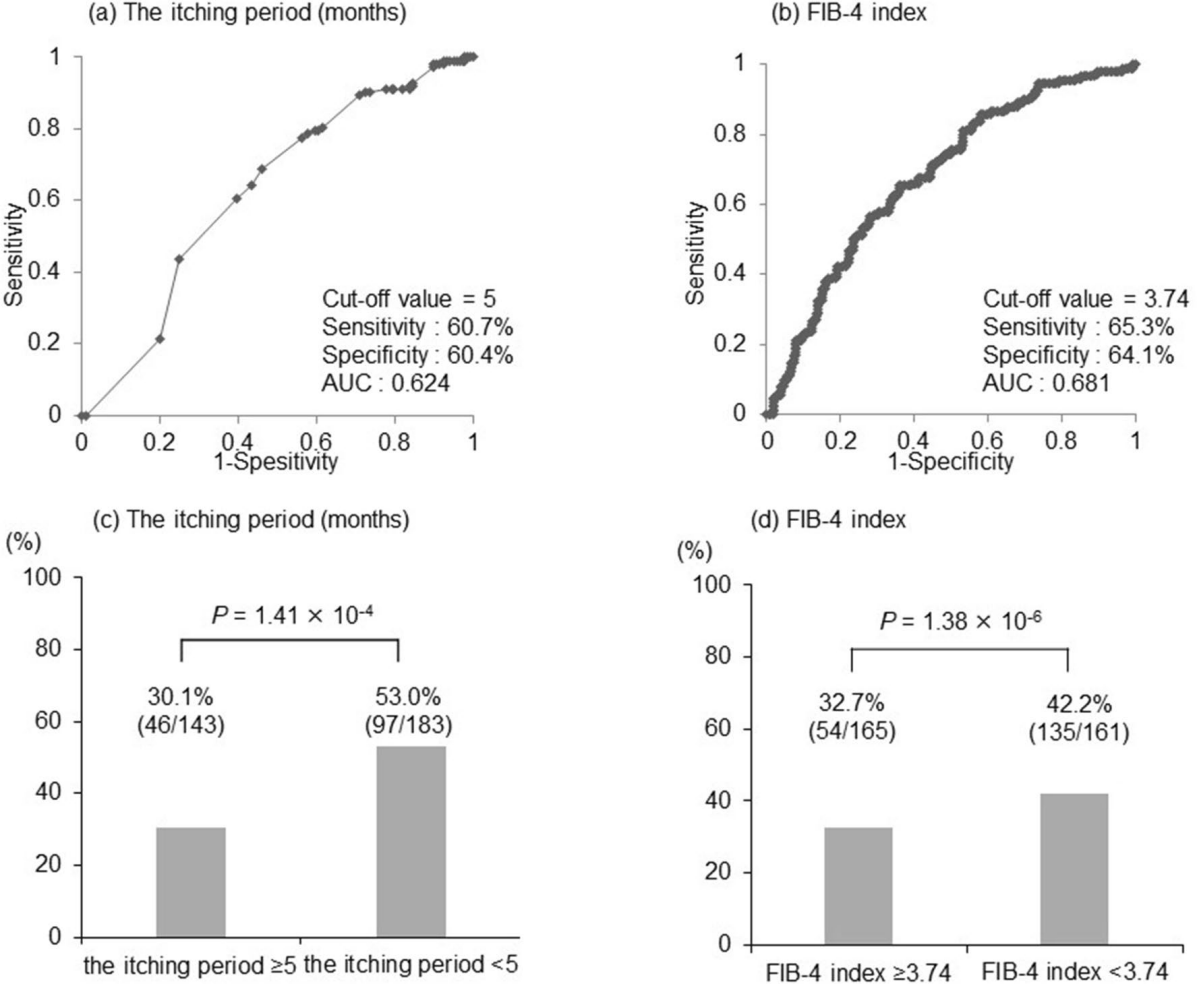
The optimal cut-off values of the itching period and FIB-4 index and the remarkable response rates. (**a**,**b**) The optimal cut-off values on the ROC curves in predicting remarkable response were 5 months for the itching period and 3.74 for FIB-4 index. (**c**,**d**) The remarkable response rates were 30.1% vs. 53.0% in patients with the itching period of ≥ 5 vs. < 5 months (*p* = 1.41 × 10^−4^) and 32.7% vs. 42.2% in patients with FIB-4 index of ≥ 3.74 vs < 3.74 (*p* = 1.38 × 10^−6^).

On univariate analysis, the itching period (*p* = 2.70 × 10^−3^), Child–Pugh A or B (*p* = 2.17 × 10^−2^), platelet count (*p* = 2.28 × 10^−4^), prothrombin time (*p* = 5.58 × 10^−4^), FIB-4 index (*p* = 8.07 × 10^−6^), and ALBI score (*p* = 2.02 × 10^−4^) were significantly associated with remarkable response. On multivariate analysis, the itching period (odds ratio, 0.96; *p* = 2.10 × 10^−3^) and FIB-4 index (odds ratio, 0.83; *p* = 2.08 × 10^−3^) were extracted as the independent factors (Table 2). In the ROC curve analysis, the optimal cut-off values of the itching period and FIB-4 index for predicting remarkable response were 5 months (sensitivity 60.7%, specificity 60.4%, AUC 0.624; Fig. 2a) and 3.74 (sensitivity 65.3%, specificity 64.1%, AUC 0.681; Fig. 2b), respectively. The response rates were 30.1% vs. 53.0% in patients with the itching period of ≥ 5 vs. < 5 months (*p* = 1.41 × 10^−4^; Fig. 2c) and 32.7% vs. 42.2% in patients with FIB-4 index of ≥ 3.74 vs. < 3.74 (*p* = 1.38 × 10^−6^; Fig. 2d). No differences in the response rates were noted between nalfurafine hydrochloride alone and concurrent treatment with other conventional agents (Supplementary Fig. 4).

**Table 2:** Univariate and multiple logistic regression analyses of factors associated with remarkable response to nalfurafine hydrochloride.

Factor	Univariate analysis			Multivariate analysis		
	OR	95%CI	*P* value	OR	95%CI	*P* value
Age	0.99	0.97–1.01	0.44			
Body weight	0.99	0.98–1.02	0.96			
Itching period	0.96	0.93–0.99	2.70 × 10^–3^	0.96	0.92–0.99	2.10 × 10^–3^
Baseline VAS	1.01	0.99–1.03	0.24			
Child–Pugh class C (vs. A and B)	0.46	0.24–0.89	2.17 × 10^–2^			
Hepatocellular carcinoma (presence)	1.50	0.85–2.63	0.16			
Platelet	1.05	1.03–1.09	2.28 × 10^–4^			
PT	1.02	1.00–1.03	5.58 × 10^–4^			
AST	1.00	0.99–1.00	0.23			
ALT	1.00	0.99–1.00	0.99			
Total bilirubin	0.97	0.91–1.05	0.48			
ALP	1.00	0.99–1.00	0.66			
Creatinine	1.10	0.95–1.28	0.19			
FIB-4 index	0.84	0.77–0.91	8.07 × 10^–6^	0.83	0.81–0.99	2.08 × 10^–3^
ALBI score	0.57	0.43–0.77	2.02 × 10^–4^			

*OR* odds ratio, *CI* confidence interval, *VAS* Visual Analog Scale, *AST* aspartate aminotransferase, *ALT* alanine aminotransferase, *ALP* alkaline phosphatase, *FIB-4* fibrosis-4.

### Factors associated with relevant response to nalfurafine hydrochloride

Of the 326 patients, 222 (68.1%) had relevant response. At baseline, relevant responders showed a shorter itching period (*p* = 9.01 × 10^−4^) and Child–Pugh A or B (*p* = 3.38 × 10^−3^) as compared to non-responders (Supplementary Table 3). No significant difference was noted in other baseline factors between the two groups. On univariate analysis, the itching period (*p* = 1.15 × 10^−2^), Child–Pugh A or B (*p* = 3.47 × 10^−2^), and total bilirubin (*p* = 2.25 × 10^−2^) were significantly associated with relevant response. On multivariate analysis, the itching period (odds ratio, 0.95; *p* = 2.95 × 10^−3^) was extracted as the only independent factor (Table 3).

**Table 3:** Univariate and multiple logistic regression analyses of factors associated with relevant response to nalfurafine hydrochloride.

Factor	Univariate analysis			Multivariate analysis		
	OR	95%CI	*P* value	OR	95%CI	*P* value
Age	1.00	0.98–1.02	0.44			
Body weight	1.01	0.99–1.03	0.96			
Itching period	0.97	0.96–0.99	1.15 × 10^–2^	0.95	0.93–0.98	2.95 × 10^–3^
Baseline VAS	1.01	0.99–1.03	0.46			
Child–Pugh Class C (vs. A and B)	0.40	0.22–0.74	3.47 × 10^–2^			
Hepatocellular carcinoma (presence)	1.36	0.73–2.54	0.34			
Platelet	1.01	0.98–1.04	0.37			
PT	1.00	0.99–1.01	0.73			
AST	0.99	0.98–1.00	0.07			
ALT	1.00	0.99–1.00	0.26			
Total bilirubin	0.91	0.85–0.99	2.25 × 10^–2^			
Creatinine	1.06	0.91–1.23	0.46			
FIB-4 index	0.95	0.90–1.01	0.09			
ALBI score	0.78	0.59–1.05	0.10			

*OR* odds ratio, *CI* confidence interval, *VAS* Visual Analog Scale, *AST* aspartate aminotransferase, *ALT* alanine aminotransferase, *FIB-4* fibrosis-4.

### Effect of nalfurafine hydrochloride dose escalation

**Figure 3:**
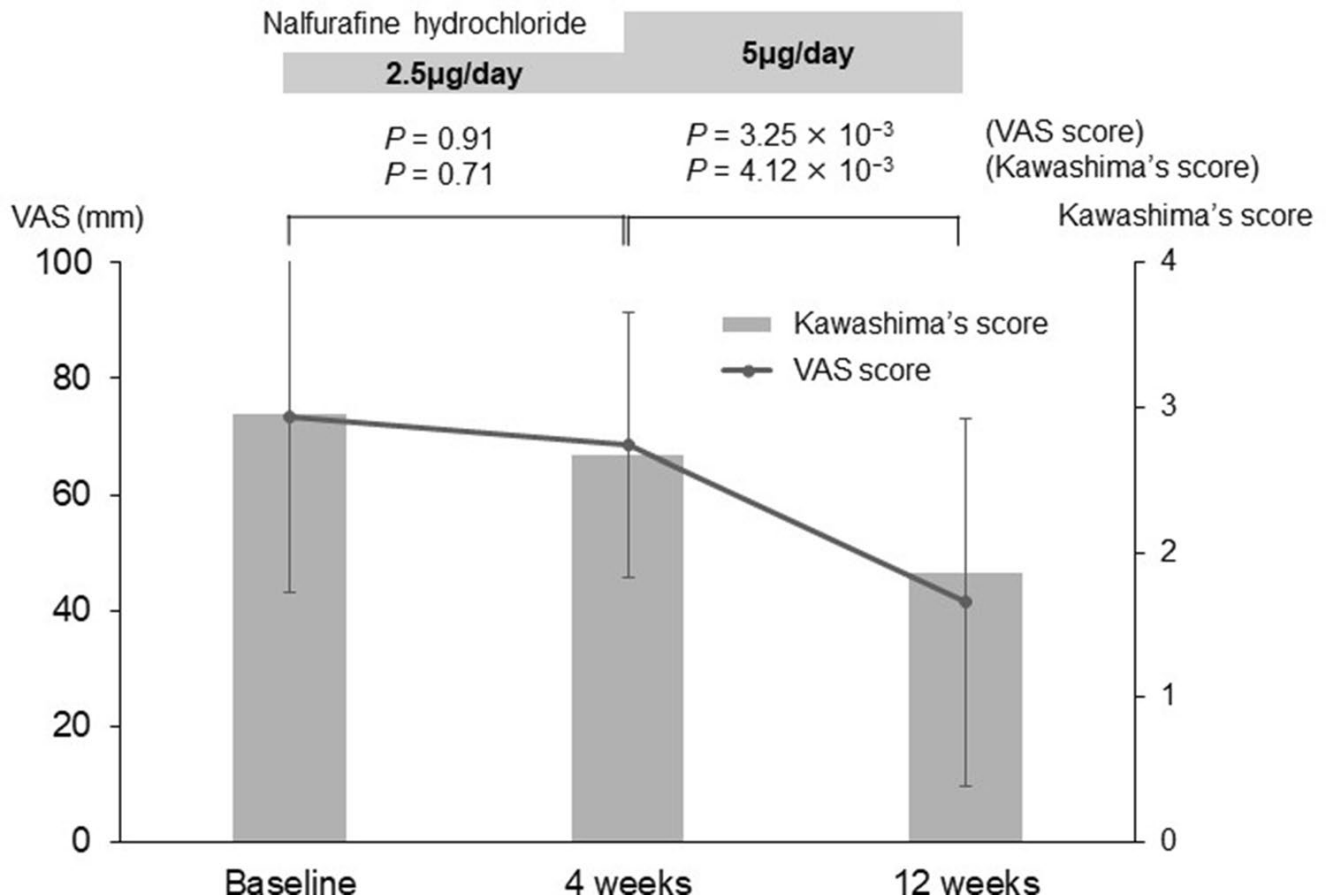
Changes in the visual analog sale score and Kawashima score of pruritus severity after nalfurafine hydrochloride in patients with dose escalation at 4 weeks.

Of the 326 patients, 104 (31.9%) had no response. Of the 104 non-responders, 11 had exacerbation despite nalfurafine hydrochloride administration (Fig. 1). There were no significant baseline factors between non-responders with and without exacerbation (n = 11 and 93, respectively; Supplementary Table 4). The dose escalation (from 2.5 to 5.0 μg/day at 4 weeks of treatment) was performed in 24 patients. These 24 patients showed a longer itching period (*p* = 2.30 × 10^−2^), Child–Pugh C (*p* = 3.37 × 10^−2^), lower platelet count (*p* = 7.45 × 10^−5^), lower serum albumin (*p* = 2.41 × 10^−3^), higher total bilirubin (*p* = 1.30 × 10^−2^), higher FIB-4 index (*p* = 9.33 × 10^−6^), and higher ALBI score (*p* = 5.55 × 10^−4^), as compared to the remaining 302 patients without dose escalation (Supplementary Table 5). Of note, the dose escalation significantly improved the VAS and Kawashima scores (*p* = 3.25 × 10^−3^ and 4.12 × 10^−3^, respectively; Fig. 3). Of the 24 patients with dose escalation, 16, 8, and 8 had relevant response, remarkable response, and non-response, respectively (Fig. 1). There were no significant factors between patients with remarkable response and non-remarkable response (Supplementary Table 6).

### Adverse events

During the observation period, nalfurafine hydrochloride was discontinued due to the following adverse events within 4 weeks (Fig. 1, Supplementary Table 1): 3 flutter (0.8%), 3 renal dysfunction (0.8%), 2 nausea (0.5%), 1 abdominal pain (0.2%), and 1 eosinophilia (0.2%). Eleven patients died of the following causes: 4 hepatocellular carcinoma, 3 liver failure, 3 gastrointestinal bleeding, and 1 septic shock. These deaths were related to aggravation of underlying advanced liver disease, but not nalfurafine hydrochloride. All adverse events and deaths occurred in patients who received nalfurafine hydrochloride 2.5 μg/day. No other serious adverse events were observed.

## Discussion

Patients with chronic liver disease are frequently accompanied by pruritus. Pruritus is usually treated with antihistamine, antiallergic drugs, and topical steroids^9^. These treatments mainly mitigate peripheral (but not central) pruritus symptoms; therefore, their effects are not always sufficient for patients with central pruritus^10^. In such patients, β-endorphin relatively strongly activates the μ-opioid receptor compared to dynorphin (which activates the κ-opioid receptor), resulting in a predominance of the pruritus-inducing μ-opioid system over the pruritic inhibitory κ-opioid system^11^. Therefore, the development of κ-opioid receptor agonists, such as nalfurafine hydrochloride, is crucial for prevailing over the pruritus-inducing μ-opioid system. Previous studies mainly reported the prevalence of pruritus in patients with chronic liver disease and the effect of nalfurafine hydrochloride and the treatment response rates in those with pruritus^6,8^. However, factors influencing the treatment response have not been evaluated and thus remains unclear. In addition, patients with Child–Pugh class C have been excluded from the study subjects. This study is the first report to identify the factors related to the response to nalfurafine hydrochloride in a relatively large, real-world cohort, including patients with Child–Pugh class C.

The liver may play a major role in the inactivation of blood-borne opioid peptides, which comprise eight or fewer amino acids and are inactivated largely by enzymatic degradation. Plasma levels of opioid peptides are likely to increase in patients with liver disease. Plasma β-endorphin levels were reported to increase in patients with primary biliary cholangitis accompanied by pruritus, and itch sensation was more severe in those with liver cirrhosis^12,13^. Patients with ascites showed the highest plasma level of Met-enkephalin, an endogenous opioid activating the δ and μ receptors, compared to those without ascites or liver cirrhosis and healthy controls^14^. In patients with chronic hepatitis C, high liver stiffness measurement was associated with pruritus even after successful anti-viral treatment^15^. These findings suggest that advanced liver fibrosis and poor liver functional reserve lead to the elevation of plasma levels of endogenous opioids (such as β-endorphin and Met-enkephalin), which could promote activation of the μ-opioid receptor in the itch-inducing system.

Although this study did not evaluate plasma levels of endogenous opioids, comparison between the groups and univariate/multivariate analysis identified Child–Pugh class A or B, higher levels of platelet count and prothrombin time, and lower levels of total bilirubin, FIB-4 index, and ALBI score as significant factors associated with the treatment responses. These analysis results suggest that patients with less-advanced liver fibrosis and better liver functional reserve have the likelihood of achieving remarkable response and relevant response to nalfurafine hydrochloride.

In a randomized, double-blinded study in Japan^7^, there was no difference in the remarkable response rate to nalfurafine hydrochloride between the 2.5 and 5.0 μg/day groups (43.8% and 44.4%, respectively). Considering that this randomized controlled study excluded patients with advanced disease stage (i.e., Child–Pugh class C) from the subjects, the dose of 2.5 μg/day of nalfurafine hydrochloride may be sufficiently effective for patients with less-advanced disease stage. In this study, patients with advanced liver fibrosis and poor liver functional reserve were likely to be resistant to the initial dose of nalfurafine hydrochloride (2.5 μg/day); however, the dose escalation to 5.0 μg/day improved low dose-resistant symptoms without any adverse events. Therefore, nalfurafine hydrochloride is initiated with the dose of 2.5 μg/day. When pruritus is not adequately improved at 4 weeks of treatment, the dose escalation to 5.0 μg/day should be adopted, especially in patients with advanced liver fibrosis and poor liver functional reserve.

In patients suffering from discontinuous pruritus, itching sensation is inhibited through the inhibitory local circuits in the spinal cord and brain by scratching the skin and stimulating the pain receptors^16,17^. Therefore, scratching the skin seems to be “rewarded” for inhibiting pruritus. Meanwhile, in patients suffering from continuous pruritus (such as those with chronic liver disease), itching sensation is not inhibited by scratching the skin and stimulating the pain receptors, because pain is perceived as pruritus through central sensitization. Although central sensitization to pruritus have not been fully clarified yet, it is reported that a broad overlap of the receptor systems exists between pain and itch, and therefore, repeated short intervals of peripheral pain stimulation result in the perception as pruritus^18,19^. In patients who fall into this condition, unrestrained scratching persistently induces the inflammatory cytokine release, resulting in the aggravation of inflammation. Furthermore, peripheral pain stimulation activates the striatum and limbic regions of the cortex, the reward and motivation centers^20^. In turn, this augmented inflammatory reaction further exacerbates pruritus, leading to a vicious cycle of itch and scratching (so-called “itch-scratch cycle”)^21^. In this study, a short itching period was identified as a significantly independent or the strongest factor associated with the treatment responses, suggesting that nalfurafine hydrochloride should be administered as early as possible when pruritus occurs. Early intervention with nalfurafine hydrochloride may disrupt the “itch-scratch cycle” and alleviate continuous central pruritus.

There are limitations of this study. First, the duration of the evaluation period was only 12 weeks, so that the long-term effect of nalfurafine hydrochloride was unclear. Second, the influence of the season on the treatment effect was not evaluated. Seasonal changes in pruritus symptoms due to the aggravating factors, such as skin stimulation by sweating in summer and dry skin in winter, have been reported^4^. Third, chemical mediators/substances, which promote or inhibit pruritus, were not investigated. Fourth, the improvement of pruritus-related symptoms (such as quality of life and sleep) with the treatment was not assessed. Finally, the sample size might be relatively small to clarify the factors associated with response to nalfurafine hydrochloride in patients with chronic liver disease.

In conclusion, this study confirms that nalfurafine hydrochloride treatment markedly and safely improves pruritus in patients with chronic liver disease. A short pruritus period, less-advanced fibrosis, and better liver functional reserve were associated with response to nalfurafine hydrochloride; therefore, early therapeutic intervention could improve pruritus more efficiently. It is necessary to pay attention to pruritus in patients with chronic liver disease, especially those with advanced disease stage, in clinical practice.

## Materials and methods

### Subjects

Between May 2015 and May 2021, 347 patients with chronic liver disease received nalfurafine hydrochloride for refractory pruritus in the 17 participating centers. Pruritus was graded based on the VAS score^22^ and the scoring system established by Kawashima et al.^23^. The patients were interviewed directly or in writing about their treatment history for pruritus, and the degree of improvement of symptoms and adverse effects in previous treatment. Refractory pruritus was defined as an inadequate effect of skin care (such as moisturizing) and/or existing treatment methods (such as antihistamine, antiallergic drugs, and topical steroids); VAS score of ≥ 25 or ≥ 50 mm; or Kawashima’s pruritus score of ≥ 1 or ≥ 2 despite the treatment. The degree of liver fibrosis was assessed using the FIB-4 index^24^. Liver functional reserve was evaluated based on the albumin–bilirubin (ALBI) grade^25^. Exclusion criteria included: (1) atopic dermatitis, chronic urticaria, or organic skin disease with generalized itching, which may be difficult to assess itching caused by chronic liver disease; (2) complications of obstructive jaundice; (3) depression, schizophrenia, or dementia; (4) drug addiction or alcohol dependence; and (5) poor adherence to the medication or drug withdrawal. This study was approved by the Nippon Medical School Hospital Ethics Committee (IRB number: B-2020-277). All patients provided written informed consent prior to study participation. The study protocol was executed in conformity to the ethical principles in the Declaration of Helsinki and the ethical guidelines those of medical and health research involving humans of the Ministry of Health, Labour and Welfare.

### Nalfurafine hydrochloride administration protocol and definition of the effect

Nalfurafine hydrochloride 2.5 μg (REMITCH CAPSULES, Toray Industries or Sumitomo Dainippon Pharma, Tokyo, Japan) was orally administered once daily after an evening meal or before the bedtime for 12 weeks. The dose could be increased from 2.5 to 5.0 μg (once daily) at 4 weeks when a patient’s complaint was poignant or pruritus deteriorated despite nalfurafine hydrochloride treatment. The degree of pruritus in the daytime and nighttime was quantitated or graded using the VAS score^22^ and Kawashima’s pruritus scoring system^23^. The VAS score ranges from 0 mm (no pruritus) to 100 mm (maximum pruritus)^22^. The Kawashima’s pruritus score comprises the five-step evaluation, ranging from score 0 (almost no pruritus) to score 4 (very severe, unbearable pruritus, sleep disturbance by pruritus)^23^. Response to nalfurafine hydrochloride at 12 weeks of treatment was defined according to a placebo-controlled double-blind phase 3 study in Japan^7^ as follows: “relevant response” was defined as a decrease in the VAS score of ≥ 25 mm (represented by the mean of the scores recorded in the daytime and nighttime) or the Kawashima’s pruritus score of ≥ 1 (represented by the greater score recorded in the daytime and nighttime); and “remarkable responder” as the VAS score of ≥ 50 mm or the Kawashima’s pruritus score of ≥ 2. Conversely, “non-response” was defined as a decrease in the VAS score of < 25 mm or the Kawashima’s pruritus score of < 1 score. When pruritus was aggravated, the condition was designated as “exacerbation.” In all patients, the degrees of pruritus were collected from the patients at the initiation of nalfurafine hydrochloride, at the time of visit 4 and 12 weeks after administration, and information was obtained from the medical record containing the results.

### Statistical analysis

Changes in the VAS and Kawashima’s pruritus score after nalfurafine hydrochloride administration were evaluated using the Friedman test, followed by post-hoc pairwise comparisons with the Bonferroni test. The two groups were compared using the Mann–Whitney *U* test. Categorical data were analyzed using the Fisher’s exact test. Correlations between continuous variables were analyzed using the Spearman’s rank correlation test. The cut-off value of FIB-4 index for predicting treatment response was calculated using a receiver operating characteristic (ROC) curve. The cut-off value for each non-invasive test was determined corresponding to the point on the area under the curve (AUC), which was closest to the upper-left corner of the plot. A *p*-value < 0.05 was regarded as significant. Statistical analysis was performed using Excel Statistics 2015 (SSRI, Tokyo).

## Supplementary Information

Supplementary Figure 1.

Supplementary Figure 2.

Supplementary Figure 3.

Supplementary Figure 4.

Supplementary Table 1.

Supplementary Table 2.

Supplementary Table 3.

Supplementary Table 4.

Supplementary Table 5.

Supplementary Table 6.

## Data Availability

All data generated or analyzed during this study are included in this published article.
