# Supplementary figures and images for: Shorter pruritus period and milder disease stage are associated with response to nalfurafine hydrochloride in patients with chronic liver disease
Source: Sci Rep. 2022 May 4;12:7311. doi: 10.1038/s41598-022-11431-1 (PMC9068920; doi:10.1038/s41598-022-11431-1)

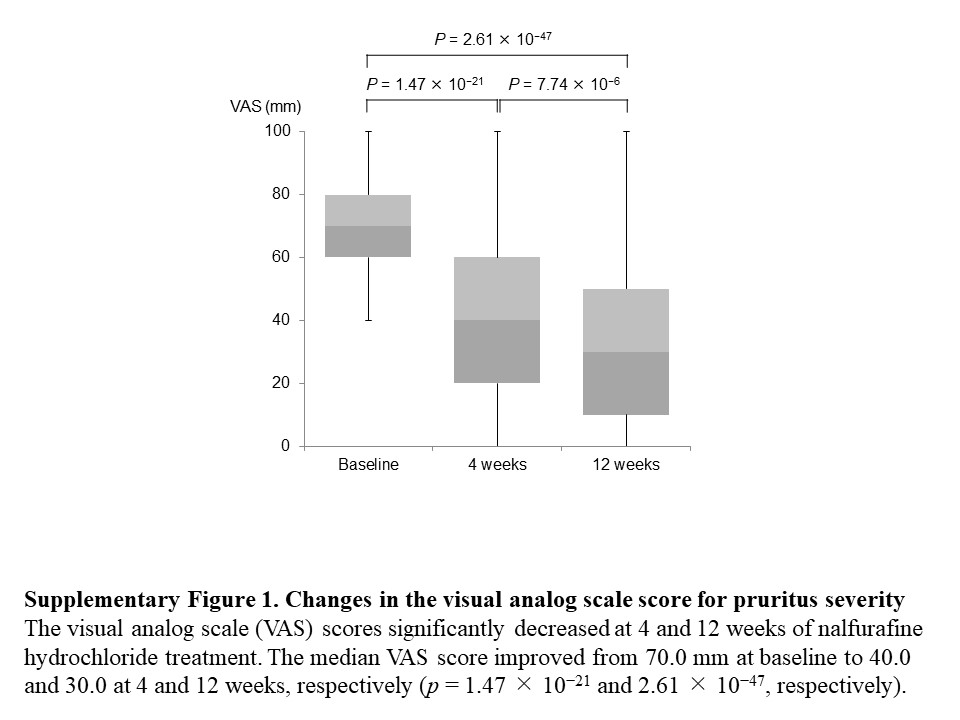

Supplement: Supplementary file 1 — Supplementary Figure 1. [file 41598_2022_11431_MOESM1_ESM.jpg]

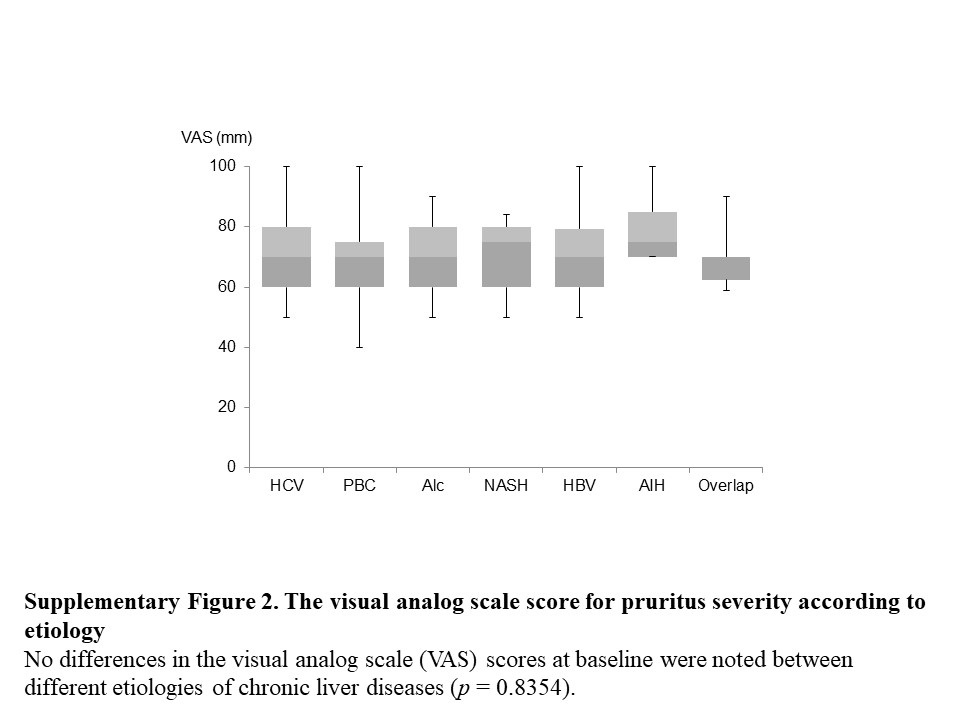

Supplement: Supplementary file 2 — Supplementary Figure 2. [file 41598_2022_11431_MOESM2_ESM.jpg]

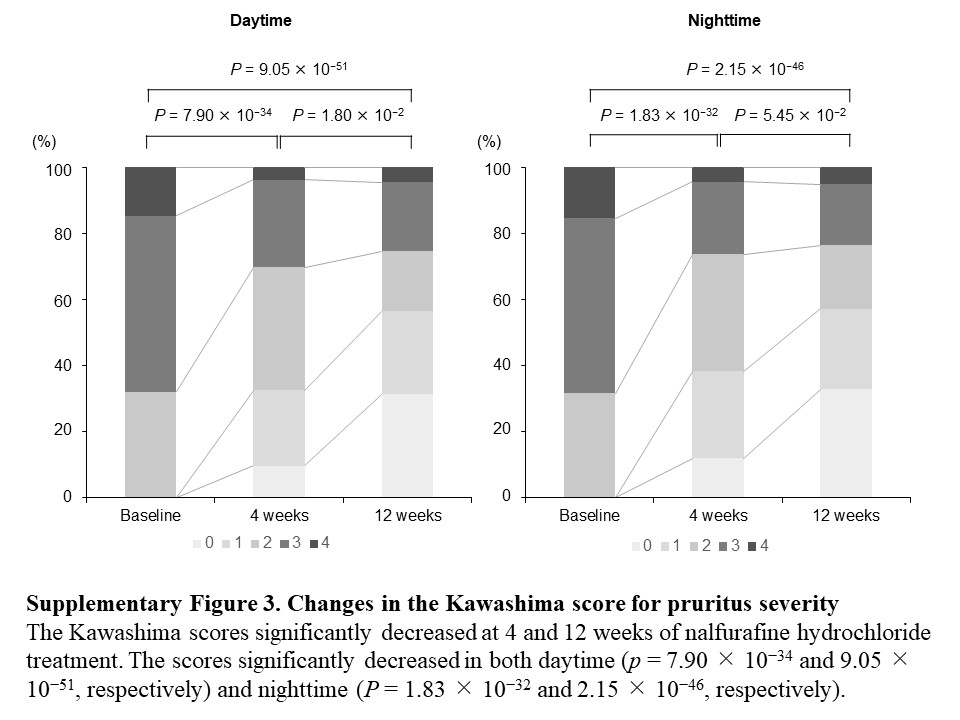

Supplement: Supplementary file 3 — Supplementary Figure 3. [file 41598_2022_11431_MOESM3_ESM.jpg]

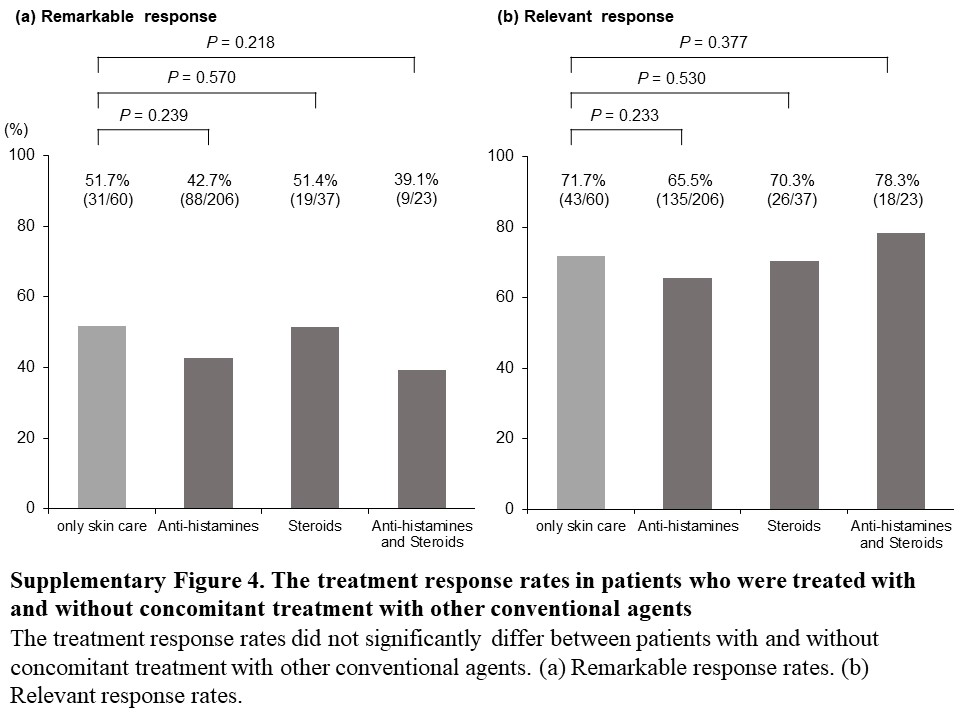

Supplement: Supplementary file 4 — Supplementary Figure 4. [file 41598_2022_11431_MOESM4_ESM.jpg]
