# Supplementary material for: Shorter pruritus period and milder disease stage are associated with response to nalfurafine hydrochloride in patients with chronic liver disease
Source: Sci Rep. 2022 May 4;12:7311. doi: 10.1038/s41598-022-11431-1 (PMC9068920; doi:10.1038/s41598-022-11431-1)
Supplement: Supplementary file 5 — Supplementary Table 1. [file 41598_2022_11431_MOESM5_ESM.docx]

Supplementary Table 1. Characteristics of 21 patients who discontinued nalfurafine hydrochloride (2.5 μg/day) within the first 4 weeks due to adverse events

| Patient | Gender | Age | Adverse event | Number (%) | Onset (day) | Relationship to nalfurafine hydrochloride |
| --- | --- | --- | --- | --- | --- | --- |
| 1 | Male | 85 | Flutter | 3 (0.8) | 14 | Probable |
| 2 | Female | 68 |  |  | NA | Probable |
| 3 | Male | 74 |  |  | 7 | Probable |
| 4 | Male | 83 | Renal dysfunction | 3 (0.8) | 28 | Probable |
| 5 | Male | 68 |  |  | NA | Probable |
| 6 | Female | 59 |  |  | NA | Probable |
| 7 | Male | 53 | Nausea | 2 (0.5) | 5 | Probable |
| 8 | Male | 74 |  |  | NA | Probable |
| 9 | Male | 68 | Abdominal pain | 1 (0.2) | 10 | Probable |
| 10 | Female | 69 | Eosinophilia | 1 (0.2) | 28 | Probable |
| 11 | Female | 45 | Death  (Hepatocellular carcinoma) | 4 (1.2) | 28 | Unrelated |
| 12 | Female | 65 |  |  | NA | Unrelated |
| 13 | Male | 89 |  |  | 14 | Unrelated |
| 14 | Male | 82 |  |  | NA | Unrelated |
| 15 | Male | 75 | Death  (Liver failure) | 3 (0.8) | NA | Unrelated |
| 16 | Male | 76 |  |  | NA | Unrelated |
| 17 | Female | 74 |  |  | NA | Unrelated |
| 18 | Male | 88 | Death  (Gastrointestinal bleeding) | 3 (0.8) | 7 | Unrelated |
| 19 | Male | 63 |  |  | NA | Unrelated |
| 20 | Female | 81 |  |  | NA | Unrelated |
| 21 | Male | 85 | Death  (Septic shock) | 1 (0.2) | 20 | Unrelated |

　　NA, not available. Total number of participating patients = 347.
