# Supplementary material for: Shorter pruritus period and milder disease stage are associated with response to nalfurafine hydrochloride in patients with chronic liver disease
Source: Sci Rep. 2022 May 4;12:7311. doi: 10.1038/s41598-022-11431-1 (PMC9068920; doi:10.1038/s41598-022-11431-1)
Supplement: Supplementary file 6 — Supplementary Table 2. [file 41598_2022_11431_MOESM6_ESM.docx]

Supplementary Table 2. Comparison of baseline characteristics between remarkable responders and non-remarkable responders

| Factor | Remarkable responders  n = 147 | Non-remarkable　responders  n = 179 | *P* value |
| --- | --- | --- | --- |
| Gender (Male/ Female) | 77/ 70 | 87/ 92 | 0.50 |
| Age (years) | 71 (37–93) | 72 (18–90) | 0.18 |
| Height (cm) | 159 (136–174) | 157 (133–176) | 0.29 |
| Body weight (kg) | 58.3 (33–89.2) | 56.3 (29.0–102.5) | 0.58 |
| Itching period (month) | 3 (1.0–72) | 6 (0.25–120) | 1.29 × 10^-5^ |
| Baseline VAS | 70 (40–100) | 70 (40–100) | 0.17 |
| Child-Pugh classification (A and B/ C) | 132/ 15 | 144/ 35 | 6.31 × 10^-3^ |
| Hepatocellular carcinoma (presence/ absence) | 32/ 115 | 28/ 151 | 0.16 |
| Platelet (×10^3^/mm^3^) | 148 (38–458) | 108 (23–549) | 1.88 × 10^-6^ |
| PT (%) | 86 (36.7–141) | 78 (6.7–173) | 6.68 × 10^-4^ |
| Albumin (g/dL) | 3.8 (1.5–4.8) | 3.3 (1.7–4.7) | 0.75 |
| AST (U/L) | 32 (3–256) | 40 (9–1177) | 4.01 × 10^-3^ |
| ALT (U/L) | 23 (2–398) | 28 (6–1509) | 0.12 |
| Total bilirubin (mg/dL) | 0.8 (0.2–21.5) | 1.1 (0.2–29) | 2.41 × 10^-2^ |
| ALP (U/L) | 45 (9–1769) | 409 (94–4600) | 0.27 |
| γ-GTP (mg/dL) | 190 (9–423) | 49 (10–1251) | 0.58 |
| BUN (mg/dL) | 17.4 (4.8–134.9) | 17.6 (5.6–208) | 0.26 |
| Creatinine (mg/dL) | 0.84 (0.38–11.6) | 0.86 (0.33–9.46) | 0.58 |
| eGFR (mL/min/1.73m^2^) | 61.0 (3.9–125) | 60.7 (4.1–140.2) | 0.36 |
| AFP (ng/mL) | 4.11 (36.2–2434) | 4.45 (1.0–12634) | 0.11 |
| M2BPGi (C.O.I.) | 2.32 (0.42–22.2) | 3.52 (0.66–20.1) | 0.07 |
| FIB-4 index | 3.20 (0.72–27.3) | 4.67 (0.56–18.1) | 4.01 × 10^-8^ |
| ALBI score | -2.43 (-3.36–0.24) | -2.01 (-3.52– -0.66) | 1.04 × 10^-4^ |

VAS, Visual Analog Scale; PT, prothrombin time; AST, aspartate aminotransferase; ALT, alanine aminotransferase; γ-GTP, gamma glutamyl transpeptidase; BUN, Blood urea nitrogen; eGFR, estimated glomerular filtration rate; AFP, α-fetoprotein; M2BPGi, Mac-2 binding protein glycosylation isomer; FIB-4, fibrosis-4; ALBI score, albumin-bilirubin score.
