# Supplementary material for: Shorter pruritus period and milder disease stage are associated with response to nalfurafine hydrochloride in patients with chronic liver disease
Source: Sci Rep. 2022 May 4;12:7311. doi: 10.1038/s41598-022-11431-1 (PMC9068920; doi:10.1038/s41598-022-11431-1)
Supplement: Supplementary file 7 — Supplementary Table 3. [file 41598_2022_11431_MOESM7_ESM.docx]

Supplementary Table 3. Comparison of baseline characteristics between relevant responders and non-responders

| Factor | Relevant responders  n = 222 | Non-responders  n = 104 | *P* value |
| --- | --- | --- | --- |
| Gender (Male/ Female) | 110/ 112 | 54/ 50 | 0.72 |
| Age (years) | 71 (37–93) | 72 (18–90) | 0.35 |
| Height (cm) | 158 (136–176) | 157 (133–175) | 0.83 |
| Body weight (kg) | 58 (30–102.5) | 55.3 (29.0–96.6) | 0.45 |
| Itching period (month) | 3 (0.25–88) | 6 (0.25–120) | 9.01 × 10^-4^ |
| Baseline VAS | 70 (40–100) | 70 (50–100) | 0.39 |
| Child-Pugh classification (A and B/ C) | 197/ 25 | 79/ 25 | 3.38 × 10^-3^ |
| Hepatocellular carcinoma (presence/ absence) | 44/ 178 | 16/ 88 | 0.35 |
| Platelet (×10^3^/mm^3^) | 141 (38–458) | 96.5 (23–549) | 0.31 |
| PT (%) | 82.6 (25–173) | 79 (6.7–137) | 0.55 |
| Albumin (g/dL) | 3.7 (1.6–4.7) | 3.2 (1.5–4.8) | 0.18 |
| AST (U/L) | 33 (9–343) | 43 (3–1177) | 0.32 |
| ALT (U/L) | 24 (6–398) | 31 (2–1509) | 0.98 |
| Total bilirubin (mg/dL) | 0.9 (0.2–29) | 1.3 (0.2–18.6) | 0.46 |
| ALP (U/L) | 371 (94–4600) | 409 (181–1189) | 0.57 |
| γ-GTP (mg/dL) | 45 (10–1769) | 53 (9–1251) | 0.39 |
| BUN (mg/dL) | 17.5 (4.8–208) | 16.5 (5.6–134.9) | 0.71 |
| Creatinine (mg/dL) | 0.88 (0.33–9.46) | 0.76 (0.4–6.59) | 0.81 |
| eGFR (mL/min/1.73m^2^) | 59 (4–140.2) | 60.7 (3.9–116.2) | 0.89 |
| AFP (ng/mL) | 4.11 (1.0–12634) | 4.91 (1.0–2434) | 0.12 |
| M2BPGi (C.O.I.) | 2.64 (0.42–22.2) | 3.71 (0.66–19.9) | 0.15 |
| FIB-4 index | 3.35 (0.72–27.3) | 7.00 (0.43–18.1) | 0.09 |
| ALBI score | -2.26 (-3.35–0.67) | -1.80 (-3.52– -0.21) | 0.22 |

VAS, Visual Analog Scale; PT, prothrombin time; AST, aspartate aminotransferase; ALT, alanine aminotransferase; γ-GTP, gamma glutamyl transpeptidase; BUN, Blood urea nitrogen; eGFR, estimated glomerular filtration rate; AFP, α-fetoprotein; M2BPGi, Mac-2 binding protein glycosylation isomer; FIB-4, fibrosis-4; ALBI score, albumin-bilirubin score.
