# Supplementary material for: Shorter pruritus period and milder disease stage are associated with response to nalfurafine hydrochloride in patients with chronic liver disease
Source: Sci Rep. 2022 May 4;12:7311. doi: 10.1038/s41598-022-11431-1 (PMC9068920; doi:10.1038/s41598-022-11431-1)
Supplement: Supplementary file 8 — Supplementary Table 4. [file 41598_2022_11431_MOESM8_ESM.docx]

Supplementary Table 4. Comparison of baseline characteristics between non-responders with and without exacerbation

| Factor | Exacerbation  n = 11 | Non-exacerbation  n = 93 | *P* value |
| --- | --- | --- | --- |
| Gender (Male/ Female) | 4/ 7 | 50/ 43 | 0.43 |
| Age (years) | 69 (54–87) | 73 (18–90) | 0.45 |
| Height (cm) | 157 (136–176) | 158 (133–174) | 0.54 |
| Body weight (kg) | 54.5 (30–73) | 55.4 (29.0–96.6) | 0.63 |
| Itching period (month) | 4 (0.25–17) | 6 (1–120) | 0.42 |
| Baseline VAS | 70 (59–80) | 70 (50–100) | 0.17 |
| Child-Pugh class (A and B/ C) | 7/ 4 | 72/ 21 | 0.20 |
| Hepatocellular carcinoma (presence/ absence) | 1/ 10 | 15/ 78 | 0.66 |
| Platelet (×10^3^/mm^3^) | 89 (40–134) | 98 (23–549) | 0.21 |
| PT (%) | 65.3 (31.9–104) | 79.7 (6.7–173) | 0.12 |
| Albumin (g/dL) | 2.9 (2.0–4.2) | 3.3 (1.5–4.8) | 0.19 |
| AST (U/L) | 49 (27–148) | 40.5 (3–1177) | 0.43 |
| ALT (U/L) | 36 (14–49) | 32 (2–1509) | 0.49 |
| Total bilirubin (mg/dL) | 1.7 (0.6–4.0) | 1.3 (0.2–18.6) | 0.25 |
| ALP (U/L) | 453 (199–1032) | 400 (181–1189) | 0.40 |
| γ-GTP (mg/dL) | 72 (19–120) | 48 (9–1251) | 0.86 |
| BUN (mg/dL) | 20.8 (13.6–134.9) | 16.3 (5.6–86.6) | 0.07 |
| Creatinine (mg/dL) | 0.96 (0.56–6.59) | 0.76 (0.41–9.46) | 0.06 |
| eGFR (mL/min/1.73m^2^) | 47.4 (7.1–84) | 67.5 (3.9–140.2) | 0.08 |
| AFP (ng/mL) | 7.87 (1.0–2434) | 4.80 (1.1–12634) | 0.15 |
| M2BPGi (C.O.I.) | 6.95 (0.85–15.3) | 3.59 (0.66–20.1) | 0.23 |
| FIB-4 index | 8.67 (4.65–17.3) | 6.90 (0.56–18.1) | 0.11 |
| ALBI score | -1.61 (-2.73 – 0.52) | -1.96 (-3.53– -0.21) | 0.22 |

VAS, Visual Analog Scale; PT, prothrombin time; AST, aspartate aminotransferase; ALT, alanine aminotransferase; γ-GTP, gamma glutamyl transpeptidase; BUN, Blood urea nitrogen; eGFR, estimated glomerular filtration rate; AFP, α-fetoprotein; M2BPGi, Mac-2 binding protein glycosylation isomer; FIB-4, fibrosis-4; ALBI score, albumin-bilirubin score.
