# Supplementary material for: Shorter pruritus period and milder disease stage are associated with response to nalfurafine hydrochloride in patients with chronic liver disease
Source: Sci Rep. 2022 May 4;12:7311. doi: 10.1038/s41598-022-11431-1 (PMC9068920; doi:10.1038/s41598-022-11431-1)
Supplement: Supplementary file 9 — Supplementary Table 5. [file 41598_2022_11431_MOESM9_ESM.docx]

Supplementary Table 5. Comparison of baseline characteristics between patients with and without dose escalation of nalfurafine hydrochloride (2.5 to 5.0 μg/day) at 4 weeks

| Factor | 2.5 μg/day n = 302 | 5.0 μg/day  n = 24 | *P* value |
| --- | --- | --- | --- |
| Gender (Male/ Female) | 150/ 152 | 10/ 6 | 0.54 |
| Age (years) | 71 (18–93) | 74 (35–87) | 0.98 |
| Height (cm) | 158 (133–176) | 160 (141–174) | 0.75 |
| Body weight (kg) | 57.1 (29.0–102) | 52.9 (38–81) | 0.82 |
| Itching period (month) | 3 (0.25–120) | 8 (1–45) | 2.30 × 10^-2^ |
| Baseline VAS | 70 (50–100) | 70 (50–100) | 0.54 |
| Child-Pugh class (A and B/ C) | 260/ 42 | 16/ 8 | 3.37 × 10^-2^ |
| Hepatocellular carcinoma (presence/ absence) | 53/ 249 | 7/ 17 | 0.31 |
| Platelet (×10^3^/mm^3^) | 152 (36–549) | 85 (23–230) | 7.45 × 10^-5^ |
| PT (%) | 82.9 (24.0–173) | 65.6 (6.7–110) | 0.06 |
| Albumin (g/dL) | 3.5 (1.5–4.8) | 2.9 (1.6–4.1) | 2.41 × 10^-3^ |
| AST (U/L) | 51 (2.9–1177) | 66 (14–343) | 0.11 |
| ALT (U/L) | 44 (2–1509) | 37 (9–158) | 0.91 |
| Total bilirubin (mg/dL) | 1.8 (0.2–29.0) | 2.2 (0.4–9.5) | 1.30 × 10^-2^ |
| ALP (U/L) | 533 (94–4600) | 455 (173–900) | 0.78 |
| γ-GTP (mg/dL) | 133 (9–1769) | 74 (14–662) | 0.11 |
| BUN (mg/dL) | 21.7 (4.8–208) | 22.5 (9.9–71.7) | 0.37 |
| Creatinine (mg/dL) | 1.31 (0.39–9.50) | 1.35 (0.51–8.42) | 0.99 |
| eGFR (mL/min/1.73m^2^) | 59.8 (3.9–140.2) | 59.3 (4.0–91) | 0.55 |
| AFP (ng/mL) | 149.6 (0.7–12634) | 15.81 (1.0–89.0) | 0.27 |
| M2BPGi (C.O.I.) | 4.92 (0.42–17.3) | 11.19 (1.20–22.21) | 0.16 |
| FIB-4 index | 4.56 (0.44–16.2) | 11.1 (1.98–27.3) | 9.33 × 10^-6^ |
| ALBI score | -2.10 (-3.52– -0.66) | -1.42 (-2.68–0.61) | 5.55 × 10^-4^ |

VAS, Visual Analog Scale; PT, prothrombin time; AST, aspartate aminotransferase; ALT, alanine aminotransferase; γ-GTP, gamma glutamyl transpeptidase; BUN, Blood urea nitrogen; eGFR, estimated glomerular filtration rate; AFP, α-fetoprotein; M2BPGi, Mac-2 binding protein glycosylation isomer; FIB-4, fibrosis-4; ALBI score, albumin-bilirubin score.
