# Supplementary material for: Shorter pruritus period and milder disease stage are associated with response to nalfurafine hydrochloride in patients with chronic liver disease
Source: Sci Rep. 2022 May 4;12:7311. doi: 10.1038/s41598-022-11431-1 (PMC9068920; doi:10.1038/s41598-022-11431-1)
Supplement: Supplementary file 10 — Supplementary Table 6. [file 41598_2022_11431_MOESM10_ESM.docx]

Supplementary Table 6. Comparison of baseline characteristics between remarkable responders and non-remarkable responders, who escalated a dose of nalfurafine hydrochloride to 5.0 μg/day at 4 weeks

| Factor | Remarkable responders  n = 8 | Non-remarkable responders  n = 16 | *P* value |
| --- | --- | --- | --- |
| Gender (Male/ Female) | 7/ 1 | 7/ 9 | 0.06 |
| Age (years) | 75.5 (51–85) | 71 (35–87) | 0.83 |
| Height (cm) | 163 (150–173) | 159 (141–174) | 0.46 |
| Body weight (kg) | 62.2 (38–75.1) | 52.8 (44.4–81) | 0.78 |
| Itching period (month) | 4.0 (1.0–24) | 12 (3.0–45) | 0.74 |
| Baseline VAS | 70 (50–90) | 70 (50–100) | 0.84 |
| Child-Pugh class (A and B/ C) | 11/ 5 | 5/ 3 | 0.46 |
| Hepatocellular carcinoma (presence/ absence) | 4/ 4 | 12/ 4 | 0.53 |
| Platelet (×10^3^/mm^3^) | 74 (38–128) | 91 (23–230) | 0.57 |
| PT (%) | 68.6 (47.0–84.0) | 64,2 (6.7–110) | 0.53 |
| Albumin (g/dL) | 2.7 (1.6–3.7) | 3.0 (1.7–4.1) | 0.68 |
| AST (U/L) | 99 (21–343) | 49 (14–112) | 0.22 |
| ALT (U/L) | 51 (10–158) | 31 (9–78) | 0.48 |
| Total bilirubin (mg/dL) | 2.8 (0.4–9.5) | 2.0 (0.4–5.3) | 0.72 |
| ALP (U/L) | 395 (318–464) | 469 (173–900) | 0.31 |
| γ-GTP (mg/dL) | 133 (21–662) | 44 (14–143) | 0.10 |
| BUN (mg/dL) | 33.0 (10.2–71.7) | 16.9 (9.9–49.6) | 0.15 |
| Creatinine (mg/dL) | 1.31 (0.61–3.21) | 1.41 (0.51–8.41) | 0.71 |
| eGFR (mL/min/1.73m^2^) | 55.4 (16.0–91) | 61.4 (4.0–84.0) | 0.88 |
| AFP (ng/mL) | 17.9 (1.5–67.6) | 14.7 (1.0–89.0) | 0.53 |
| M2BPGi (C.O.I.) | 20.2 (18.2–22.2) | 7.57 (1.20–20.0) | 0.44 |
| FIB-4 index | 13.12 (6.03–27.28) | 10.05 (1.98–18.11) | 0.81 |
| ALBI score | -1.34 (-2.39 – -0.24) | -1.46 (-2.67–0.62) | 0.97 |

VAS, Visual Analog Scale; PT, prothrombin time; AST, aspartate aminotransferase; ALT, alanine aminotransferase; γ-GTP, gamma glutamyl transpeptidase; BUN, Blood urea nitrogen; eGFR, estimated glomerular filtration rate; AFP, α-fetoprotein; M2BPGi, Mac-2 binding protein glycosylation isomer; FIB-4, fibrosis-4; ALBI score, albumin-bilirubin score.
